# Digital self-care in the management of spine musculoskeletal disorders: A systematic review and meta-analysis

**DOI:** 10.1590/1518-8345.6423.3909

**Published:** 2023-05-12

**Authors:** Zulamar Aguiar Cargnin, Dulcinéia Ghizoni Schneider, Joanito Niquini Rosa-Junior

**Affiliations:** 1 Universidade Federal de Santa Catarina, Florianópolis, SC, Brasil

**Keywords:** Low Back Pain, Neck Pain, Back Pain, Self-Management, Pain Management, Internet, Dolor de la Región Lumbar, Dolor de Cuello, Dolor de Espalda, Automanejo, Manejo del Dolor, Internet, Dor Lombar, Dor Cervical, Dor nas Costas, Autogestão, Manejo da Dor, Internet

## Abstract

**Objective:**

to analyze the effectiveness of digital self-care in the management of pain and functional disability among people with spine musculoskeletal disorders.

**Method:**

a systematic literature review, developed with the PRISMA checklist, of randomized clinical trials of people with spine musculoskeletal disorders and digital interventions accessed by means of computers, smartphones or other portable devices. Databases researched: National Library of Medicine, Excerpta Médica dataBASE, SciVerse Scopus, Literatura Latino-Americana e do Caribe em Ciências da Saúde, Science Citation Indexes, Cumulative Index to Nursing and Allied Health Literature and Physiotherapy Evidence Database. The descriptive synthesis of the results and by means of meta-analyses (fixed-effects model) was performed with the Review Manager software. The methodological quality was evaluated with the Physiotherapy Evidence Database scale.

**Results:**

a total of 25 trials were selected (5,142 participants), which showed statistically significant improvements (p <0.05) in 54% (12/22) in the pain levels and 47% (10/21) in functional disability in the Intervention Group. The meta-analyses showed moderate effects on pain intensity and small effects on functional disability. There was a predominance of medium quality studies.

**Conclusion:**

the digital care interventions showed a beneficial result in pain intensity and in functional disability, mainly for chronic low back pain. Digital care emerges as promising to support self-management of the spine musculoskeletal conditions. PROSPERO registry number CRD42021282102.

## Abstract (es)

**Objetivo:**

analizar la efectividad del autocuidado digital en el manejo del dolor y la discapacidad funcional en personas con trastornos musculoesqueléticos espinales.

**Método:**

revisión sistemática de la literatura, desarrollada con la checklist PRISMA, de ensayos clínicos aleatorizados sobre personas con trastornos musculoesqueléticos de columna e intervenciones digitales a las que se accede por computadora, smartphones u otro dispositivo portátil. Bases de datos consultadas: National Library of Medicine, Excerpta Médica dataBASE, SciVerse Scopus, Literatura Latinoamericana y del Caribe en Ciencias de la Salud, Science Citation Indexes, Cummulative Index to Nursing and Allied Health Literature y Physiotherapy Evidence Database. Síntesis de resultados descriptiva y por metanálisis (modelo de efectos fijos) realizada con el software Review Manager. Calidad metodológica evaluada mediante la escala Physiotherapy Evidence Database.

**Resultados:**

se seleccionaron 25 ensayos (5142 participantes) que mostraron mejoras estadísticamente significativas (p<0,05) del 54% (12/22) en los niveles de dolor y del 47% (10/21) en la discapacidad funcional en el grupo intervención. Los metanálisis mostraron efectos moderados sobre la intensidad del dolor y efectos pequeños sobre la discapacidad funcional. Predominaron los estudios de calidad media.

**Conclusión:**

las intervenciones de atención digital demostraron resultados beneficiosos para la intensidad del dolor y la discapacidad funcional, principalmente para el dolor lumbar crónico. Se ha demostrado que la atención digital es promisoria para favorecer el automanejo de las afecciones musculoesqueléticas de columna. Registro PROSPERO CRD42021282102.

## Abstract (pt)

**Objetivo:**

analisar a efetividade do autocuidado digital no manejo da dor e incapacidade funcional em pessoas com distúrbios musculoesqueléticos de coluna.

**Método:**

revisão sistemática da literatura, desenvolvida com o checklist PRISMA, de ensaios clínicos randomizados de pessoas com distúrbios musculoesqueléticos de coluna e intervenções digitais acessadas por computador, smartphones ou outro dispositivo portátil. Bases pesquisadas: National Library of Medicine, Excerpta Médica dataBASE, SciVerse Scopus, Literatura Latino-Americana e do Caribe em Ciências da Saúde, Science Citation Indexes, Cummulative Index to Nursing and Allied Health Literature e Physiotherapy Evidence Database. Síntese dos resultados descritiva e por metanálises (modelo de efeitos fixos) com o software Review Manager. Qualidade metodológica avaliada pela escala Physiotherapy Evidence Database.

**Resultados:**

selecionaram-se 25 ensaios (5142 participantes) que revelaram melhoras estatisticamente significativas (p<0,05) em 54% (12/22) nos níveis de dor e 47% (10/21) na incapacidade funcional no grupo intervenção. As metanálises mostraram efeitos moderados na intensidade da dor e pequenos na incapacidade funcional. Houve predominância de estudos de média qualidade.

**Conclusão:**

intervenções de cuidados digitais mostraram resultado benéfico na intensidade da dor e na incapacidade funcional principalmente para dor lombar crônica. Evidenciam-se os cuidados digitais como promissores para apoiar o autogerenciamento das condições musculoesqueléticas de coluna. Registro PROSPERO CRD42021282102.

**Highlights:**

**(1)** The digital interventions were not inferior to in-person care.

**(2)** Digital care is promising to support self-management.

**(3)** There is a need to standardize the report of results in clinical trials.

**(4)** Better quality studies are required.

**(5)** Attention should be paid in strategies to support user acceptance and adherence.

## Introduction

Spine musculoskeletal disorders are considered as an important public health problem due to their high prevalence, affecting all age groups and socioeconomic levels. Their management is still a challenge due to the different causes and triggering factors. Their control is justified by the impact they cause on the person and on the increased costs related to medical care, absences from work and medical certificates ^( 1)^ . They involve a combination of multidimensional influences, both physical and psychosocial. Due to this biopsychosocial character, multidisciplinary treatment programs with physical, psychological, and educational strategies are recommended ^( 2)^ . Thus, an option for their management is the self-care model, which proposes mutual and interactive collaborations between professionals and patients. In this perspective, individuals manage the symptoms resulting from a chronic condition, that is, they have the autonomy to monitor and manage their own health in the physical, emotional and social dimensions ^( 3)^ . Digital technologies can ease education, prevention, promotion and management in terms of health ^( 4)^ . 

In its work process, the health area has required actions that adapt to the technological transformations; however, they are still insufficient and little explored. The innovations that provide answers, be them operational, managerial or supportive of decision-making, contribute to the education and care process ^( 5)^ . In the midst of the information age, programs based on information technologies and “e-health” (digital tools and solutions) are promising for the improvement of clinical processes to prevent, treat, promote and maintain health ^( 2, 4, 6)^ . They offer advantages such as easy accessibility, availability, convenience because they can be used anywhere, customability and possibility of communicating with the professionals ^( 4)^ . In addition to that, face-to-face treatments involve transporting one or more professionals to the care system at specific times, and they cannot monitor the patients’ engagement and well-being on a daily basis ^( 6)^ . 

The studies need to measure this self-management in a more effective way. In this sense, “e-health” can be a promising strategy in the improvement of clinical processes and outcomes. However, it lacks scientific evidence in terms of content and implementation and, consequently, it needs to be better evaluated ^( 7)^ . Websites, programs and apps containing diverse and reliable information that meets the consumers’ needs are still missing. More research studies are necessary to assess if knowledge improves the results and behaviors ^( 8)^ . 

Considering that spine musculoskeletal disorders are highly prevalent and contribute to functional disability, strategic interventions with accessible models are required to influence the outcome measures. In this sense, the objective is to analyze the effectiveness of digital self-care in the management of pain and functional disability among people with spine musculoskeletal disorders.

## Method

### Type of study

This is a Systematic Review (SR) of the literature, registered at the International Prospective Register of Ongoing Systematic Reviews (PROSPERO) platform (Registration number CRD42021282102) on November 19 ^th^, 2021, and developed in accordance with the recommendations set forth in the Preferred Reporting Items for Systematic Reviews and Meta-Analyses (PRISMA) checklist ^( 9)^ . Development of the review was based on the Cochrane Manual for systematic reviews, version 6.3 of 2022 ^( 10)^ . 

### Search strategy

The review question was as follows: Are digital self-care interventions effective in the management of pain and functional disability among people with spine musculoskeletal disorders? The model defined by the PICO acronym was used, namely: Population/Condition: people with spine musculoskeletal disorders. Intervention: digital self-care. Comparison: non-digital usual care intervention; non-interactive digital intervention; waiting list. Result: pain management, self-care. Elaboration of the search mechanism was aided by two librarians. The controlled descriptors were obtained via the Descriptors in Health Sciences ( *Descritores em Ciências da Saúde*, DeCS) and terms from the Medical Subject Headings (MESH). One librarian devised the search strategy and the other validated it based on the Peer Review of Electronic Search Strategies (PRESS) tool, a checklist for research strategies for validating the search strategy ^( 11)^ . The following databases were researched: US National Library of Medicine (PubMed), *Excerpta Médica dataBASE* (Embase), SciVerse Scopus (Scopus) and *Literatura Latino-Americana e do Caribe em Ciências da Saúde* (LILACS), Science Citation Indexes (Web of Science), Cumulative Index to Nursing and Allied Health Literature (CINAHL) and Physiotherapy Evidence Database (PEDro), in addition to the manual search conducted in the references of the studies ( Figure 1). 

**Figure 1 -:** Search strategies. Florianópolis, SC, Brazil, 2022

Data base	Search strategy
LILACS * (strategy in Portuguese, Spanish and English) Scopus †, Web of Science ‡, CINAHL §, Embase || (only the strategy in English)	((“Terapia assistida por computador” OR “intervenção digital” OR “intervenção baseada na Web” OR “Intervenção Baseada em Internet” OR “Intervenção Online” OR “Intervenção da Internet” OR “Terapia por exercício” OR “Exercício Terapêutico” OR “Exercício de Reabilitação” OR “saúde digital” OR “Ciber Saúde” OR “Ciber-Saúde” OR Cibersaúde OR “e-Saúde” OR “eSaúde” OR “Medicina 2.0” OR “mSaúde” OR “Saúde 2.0” OR “Saúde Conectada” OR “Saúde Digital” OR “Saúde Eletrônica” OR “Saúde Móvel” OR “Saúde Onipresente” OR “Saúde Pervasiva” OR “Saúde Ubíqua” OR Telemedicina OR “Tele-Serviços em Saúde” OR Teleassistência OR telecuidado OR Telecura OR Telessaúde OR “Telesserviços de Saúde” OR “Telesserviços em Saúde” OR “Telesserviços na Saúde” OR “uSaúde” OR internet OR telerreabilitação OR “Reabilitação à Distância” OR “aplicativo móvel” OR “Aplicativos Eletrônicos Portáteis” OR “Aplicativos de Software Portáteis” OR “Aplicativos em Dispositivos Móveis” OR “Aplicativos para Dispositivos Móveis” OR “Apps Móveis” OR “Aplicativos Móveis” OR “telefones celulares” OR Smartphone OR Smartfone OR Smartfones OR “Telefone Celular Inteligente” OR “Telefone Inteligente” OR “Telefone Móvel Inteligente” OR “Telefones Celulares Inteligentes” OR “Telefones Inteligentes” OR “Telefones Móveis Inteligente” OR “Terapia assistida por computador”) AND (“Manejo da dor” OR Autocuidado OR “Auto gerenciamento” OR “Auto Gerenciamento” OR “Auto Gestão” OR “AutoGerenciamento” OR “Auto-Gestão” OR Autogerenciamento OR Autogestão) AND (“distúrbios musculoesqueléticos de coluna” OR Cervicalgia OR “Dor Cervical” OR “Dor na Nuca” OR “Dor no Pescoço” OR “dor dorsal” OR “dor lombar” OR Lombalgia OR Lumbago OR “dor nas costas” OR Dorsopatia)) OR ((“Terapia assistida por computador” OR Internet OR “Intervención basada en la Internet” OR “terapia por ejercicio” OR “Ejercicio Terapéutico” OR “Ejercicio de Rehabilitación” OR Telemedicina OR “Medicina 2.0” OR “Ciber Salud” OR “Ciber-Salud” OR Cibersalud OR eSalud OR mSalud OR “Salud 2.0” OR “Salud Conectada” OR “Salud Digital” OR “Salud Electrónica” OR “Salud Móvil” OR “Salud Mueble” OR “Salud Omnipresente” OR “Salud Pervasiva” OR “Salud Ubicua” OR Teleasistencia OR Telecuidado OR Telecura OR Telesalud OR “Teleservicios de Salud” OR “Teleservicios Sanitarios” OR “uSalud” OR telerreabilitación OR Telerehabilitación OR “aplicación movil” OR “Aplicaciones Móviles” OR “Aplicaciones Electrónicas Portátiles” OR “Aplicaciones de Software Portátiles” OR “Teléfonos celulares” OR “Teléfono Inteligente” OR Smartfone OR Smartfones OR Smartphone OR Smartphones OR “Teléfono Celular Inteligente” OR “Teléfono Móvil Inteligente” OR “Teléfonos Celulares Inteligentes” OR “Teléfonos Inteligentes” OR “Teléfonos Móviles Inteligentes”) AND (“Manejo del dolor” OR Autocuidado OR Automanejo) AND (“trastornos musculoesqueléticos espinales” OR “Dolor de Espalda” OR “Dolor de la región lumbar” OR “Dolor de cuello” OR “Cuello Doloroso” OR “Dolor Cervical” OR Lombalgia OR Lumbago)) OR ((“Computer Assisted Therapy” OR “Computer-Assisted Therapies” OR “Computer-Assisted Therapy” OR “digital intervention” OR “Web-based Interventions” OR “Internet-Based Intervention” OR “Internet-Based Interventions” OR “Web-based Intervention” OR “Web based Intervention” OR “Online Intervention” OR “Online Interventions” OR “Internet Intervention” OR “Internet Interventions” OR “Exercise therapy” OR “Remedial Exercise” OR “Remedial Exercises” OR “Exercise Therapies” OR “Rehabilitation Exercise” OR “Rehabilitation Exercises” OR “Telemedicine” OR “e-Health” OR “Connected Health” OR “Digital Health” OR “eHealth” OR “Health 2.0” OR “Health Tele-Services” OR “Health Teleservices” OR “Medicine 2.0” OR “mHealth” OR “mHealth Alliance” OR “Mobile Health” OR “Pervasive Computing Technologies for Healthcare” OR “Pervasive Health” OR “Telecare” OR “Telecure” OR “Telehealth” OR “Teleservices in the Health Sector” OR “u-Health” OR “Ubiquitous Health” OR “Internet” OR “Telerehabilitation” OR “Telehabilitation” OR “Telerehabilitations” OR “Tele-rehabilitation” OR “Tele rehabilitation” OR “Tele-rehabilitations” OR “Remote Rehabilitation” OR “Remote Rehabilitations” OR “Virtual Rehabilitation” OR “Virtual Rehabilitations” OR “Mobile Applications” OR “Mobile App” OR “Mobile Application” OR “Mobile Apps” OR “Portable Electronic App” OR “Portable Electronic Application” OR “Portable Electronic Applications” OR “Portable Electronic Apps” OR “Portable Software App” OR “Portable Software Application” OR “Portable Software Applications” OR “Portable Software Apps” OR “Smartphone” OR “Mobile Phone” OR “Smart Phone” OR “Smart Phones” OR “Smartphones” OR “Smartphone” OR “Mobile Phone” OR “Smart Phone” OR “Smart Phones” OR “Smartphones”) AND (“Pain Management” OR “Pain Managements” OR “Self Care” OR “SelfCare” OR “Self Management” OR “Self-Management”) AND (“spinal musculoskeletal disorders” OR “neck pain” OR “Neck Ache” OR “Neck Aches” OR “Cervicalgia” OR “Cervicalgias” OR “Cervicodynia” OR “Cervicodynias” OR “Neckache” OR “Neckaches” OR “Cervical Pain” OR “Cervical Pains” OR “low back pain” OR “Back Pains” OR “Lumbago” OR “back pain” OR “Backache” OR “Backaches” OR “Back Ache” OR “Back Aches” OR “Vertebrogenic Pain Syndrome” OR “Vertebrogenic Pain Syndromes”))
PubMed¶	((“Therapy, Computer-Assisted”[Mesh] OR “Computer Assisted Therapy” [Title/Abstract] OR “Computer-Assisted Therapies”[Title/Abstract] OR “Computer-Assisted Therapy”[Title/Abstract] OR “digital intervention” [Title/Abstract] OR “Internet-Based Intervention”[Mesh] OR “Web-based Interventions”[Title/Abstract] OR “Internet-Based Intervention” [Title/Abstract] OR “Internet-Based Interventions”[Title/Abstract] OR “Web-based Intervention”[Title/Abstract] OR “Web based Intervention” [Title/Abstract] OR “Online Intervention”[Title/Abstract] OR “Online Interventions”[Title/Abstract] OR “Internet Intervention” [Title/Abstract] OR “Internet Interventions”[Title/Abstract] OR “Exercise Therapy”[Mesh] OR “Exercise therapy”[Title/Abstract] OR “Remedial Exercise”[Title/Abstract] OR “Remedial Exercises” [Title/Abstract] OR “Exercise Therapies”[Title/Abstract] OR “Rehabilitation Exercise”[Title/Abstract] OR “Rehabilitation Exercises”[Title/Abstract] OR “Telemedicine”[Mesh] OR “Telemedicine” [Title/Abstract] OR “e-Health”[Title/Abstract] OR “Connected Health” [Title/Abstract] OR “Digital Health”[Title/Abstract] OR “eHealth” [Title/Abstract] OR “Health 2.0”[Title/Abstract] OR “Health TeleServices”[Title/Abstract] OR “Health Teleservices”[Title/Abstract] OR “Medicine 2.0”[Title/Abstract] OR “mHealth”[Title/Abstract] OR “mHealth Alliance”[Title/Abstract] OR “Mobile Health”[Title/Abstract] OR “Pervasive Computing Technologies for Healthcare”[Title/Abstract] OR “Pervasive Health”[Title/Abstract] OR “Telecare”[Title/Abstract] OR “Telecure”[Title/Abstract] OR “Telehealth”[Title/Abstract] OR “Teleservices in the Health Sector”[Title/Abstract] OR “u-Health” [Title/Abstract] OR “Ubiquitous Health”[Title/Abstract] OR “Internet” [Title/Abstract] OR “Telerehabilitation”[Mesh] OR “Telerehabilitation” [Title/Abstract] OR “Telehabilitation”[Title/Abstract] OR “Telerehabilitations”[Title/Abstract] OR “Tele-rehabilitation” [Title/Abstract] OR “Tele rehabilitation”[Title/Abstract] OR “Telerehabilitations”[Title/Abstract] OR “Remote Rehabilitation” [Title/Abstract] OR “Remote Rehabilitations”[Title/Abstract] OR “Virtual Rehabilitation”[Title/Abstract] OR “Virtual Rehabilitations” [Title/Abstract] OR “Mobile Applications”[Mesh] OR “Mobile Applications”[Title/Abstract] OR “Mobile App”[Title/Abstract] OR “Mobile Application”[Title/Abstract] OR “Mobile Apps”[Title/Abstract] OR “Portable Electronic App”[Title/Abstract] OR “Portable Electronic Application”[Title/Abstract] OR “Portable Electronic Applications” [Title/Abstract] OR “Portable Electronic Apps”[Title/Abstract] OR “Portable Software App”[Title/Abstract] OR “Portable Software Application”[Title/Abstract] OR “Portable Software Applications” [Title/Abstract] OR “Portable Software Apps”[Title/Abstract] OR “Smartphone”[Mesh] OR “Smartphone”[Title/Abstract] OR “Mobile Phone” [Title/Abstract] OR “Smart Phone”[Title/Abstract] OR “Smart Phones” [Title/Abstract] OR “Smartphones”[Title/Abstract]) AND (“Pain Management”[Mesh] OR “Pain Management”[Title/Abstract] OR “Pain Managements”[Title/Abstract] OR “Self Care”[Title/Abstract] OR “SelfCare”[Title/Abstract] OR “Self Management”[Title/Abstract] OR “SelfManagement”[Title/Abstract] OR “Self Care”[Mesh] OR “Self-Management” [Mesh]) AND (“spinal musculoskeletal disorders”[Title/Abstract] OR “Neck Pain”[Mesh] OR “neck pain”[Title/Abstract] OR “Neck Ache” [Title/Abstract] OR “Neck Aches”[Title/Abstract] OR “Cervicalgia” [Title/Abstract] OR “Cervicalgias”[Title/Abstract] OR “Cervicodynia” [Title/Abstract] OR “Cervicodynias”[Title/Abstract] OR “Neckache” [Title/Abstract] OR “Neckaches”[Title/Abstract] OR “Cervical Pain” [Title/Abstract] OR “Cervical Pains”[Title/Abstract] OR “Low Back Pain”[Mesh] OR “low back pain”[Title/Abstract] OR “Back Pains” [Title/Abstract] OR “Lumbago”[Title/Abstract] OR “Back Pain”[Mesh] OR “back pain”[Title/Abstract] OR “Backache”[Title/Abstract] OR “Backaches”[Title/Abstract] OR “Back Ache”[Title/Abstract] OR “Back Aches”[Title/Abstract] OR “Vertebrogenic Pain Syndrome” [Title/Abstract] OR “Vertebrogenic Pain Syndromes”[Title/Abstract]))
PEDro **	Digital Intervention AND Back Pain

* LILACS = Literatura Latinoamericana y del Caribe en Ciencias de la Salud

† Scopus = SciVerse Scopus

‡ Web of Science = Science Citation Indexes

§ CINAHL = Cummulative Index to Nursing and Allied Health Literature

|| Embase = Excerpta Médica dataBASE

¶ PubMed = US National Library of Medicine

** PEDro = Physiotherapy Evidence Database

### Search period

The search was performed from September 2021 to February 2022 with articles in any language and with no time restrictions to monitor the technological

advances in the last decades. The EndNote software was used to manage the references.

### Selection criteria

Inclusion criteria: people over 18 years old with spine musculoskeletal disorders (neck pain, back pain or low back pain); digital interventions accessed by means of computers, smartphones or other portable devices; components of the interventions isolated or associated with health education, Cognitive Behavioral Therapy (CBT), physiotherapy and/or ergonomic guidelines. Any and all interventions in body areas other than the spine were analyzed on a case-by-case basis. The musculoskeletal condition was clinically diagnosed or defined as a report of persistent pain lasting more than three months (chronic), less than 6 weeks (acute), and from 6 to 12 weeks (subacute). This review considered all the research contexts: home, community or others, and was limited to randomized clinical trials (RCTs).

Exclusion criteria: situations in which advice is received directly from a health professional; studies of people with specific spine conditions such as spinal stenosis, post-surgery, tumors, fractures, inflammatory disorders; pregnant women; interventions with medical and/or surgical treatments or unspecified chronic pain.

### Selection process

Selection of the studies took place in three stages: analysis of the titles and abstracts; full-reading of the texts and, finally, inclusion of the studies selected in the review. Two independent reviewers selected the studies according to the eligibility criteria with reconciliation of disagreements, and a third reviewer was available in case there were any interpretation differences. The Kappa coefficient of agreement between evaluators was classified as follows: 0.0-0.20, slight agreement; 0.21-0.40, fair agreement; 0.41-0.60, moderate agreement; 0.61-0.80, substantial agreement; and 0.81-1.00, almost perfect agreement ^( 12)^ . 

### Data collection

Data extraction was carried out independently by the researchers and then compared with the aid of the Atlas Ti software, version 22, acquired through a student license for six months, which accelerated the inferential process through file management, coding and tagging of important text segments to guide and support the discussion. Subsequently, the main results of each study were organized in an Excel 2016 spreadsheet.

### Study variables

The independent variables, analyzed descriptively, listed in this study are information about the population (age, gender and Body Mass Index) and data about the studies (type of study, year of publication, country, sample size, type of intervention and characteristics, theoretical basis, spine region, instruments, adherence, monitoring, adverse events, outcomes, follow-up). The dependent variables were “pain intensity” and “functional disability” (numerical and continuous), and were analyzed in a quantitative manner by means of the meta-analyses.

### Outcomes and measuring instruments

Pain intensity was measured with scales such as the Visual Analogue Scale (VAS), the Numerical Rating Scale (NRS) and the Brief Pain Inventory or other indirect methods such as questionnaires. Functional disability was mainly measured with the Roland-Morris (RM) questionnaire and the Oswestry Disability Index (ODI).

### Data treatment and analysis

The data were analyzed descriptively and presented in figures. The meta-analyses were performed with the Review Manager 5.4.1 software (not foreseen in the protocol). The continuous numerical data of the “pain intensity” and “functional disability” outcomes were extracted with sample size, mean scores and standard deviations (SDs). When the SDs were not available, they were estimated based on the confidence intervals or on the standard errors, or extracted based on graphs available in the articles. Statistic heterogeneity for the “pain intensity” and “functional disability” variables was calculated by means of the chi-square and I ^2^ tests. The random effect model was applied in the presence of high heterogeneity (I ^2^>50%). If I ^2^ < 50% and p > 0.10, a fixed-effect model was used. Regarding the effect size, the grouped standardized mean differences (SMDs) were considered and values below 0.2 were interpreted as small effect, between 0.2 and 0.5, as moderate effect and > 0.5, as large effect ^( 4)^ . 95% confidence intervals (95% CIs) were considered. Sensitivity analyses were performed to assess stability of the results and detect the potential heterogeneity source. 

Negative values of the estimated mean difference represent an effect in favor of the Intervention Group. Analyses of subgroups considered combinable and homogeneous in relation to period, spine region, outcome measure and technology employed were performed. For three-arm RCTs, the data from the intervention and control groups were extracted. The standardized difference was employed when different scales were used for the same outcome. The results were presented by means of Forest Plots and funnel charts.

### Quality assessment

The methodological quality was assessed with the PEDro scale (not foreseen in the protocol), for being a scoring system for the general evaluation of the study. It contains eleven items, 10 of which are scored with one (1) point and indicate presence of the quality indicator and zero (0) meaning that they do not contain information or do not meet the quality indicator condition. Criteria 2-9 (random allocation, concealed allocation, baseline comparability, blinded individuals, blinded evaluators, adequate monitoring, intention-to-treat analysis) refer to the internal validity, and criteria 10-11 (comparison between groups and specific estimates and variability) refer to the statistical information. Item 1 is not considered for the final score because it assesses the external validity of the study. This scale is based on the Delphi list developed in the Netherlands and prepared by the PEDro database that evaluates the methodological quality of all the clinical trials to guide clinical decision-making. In addition to that, the studies are numbered in order of methodological importance to ease rapid access to the most valid scientific evidence possible when searching the database. It has moderate reliability among the evaluators ^( 13)^ . 

## Results

**Figura 2 -:**
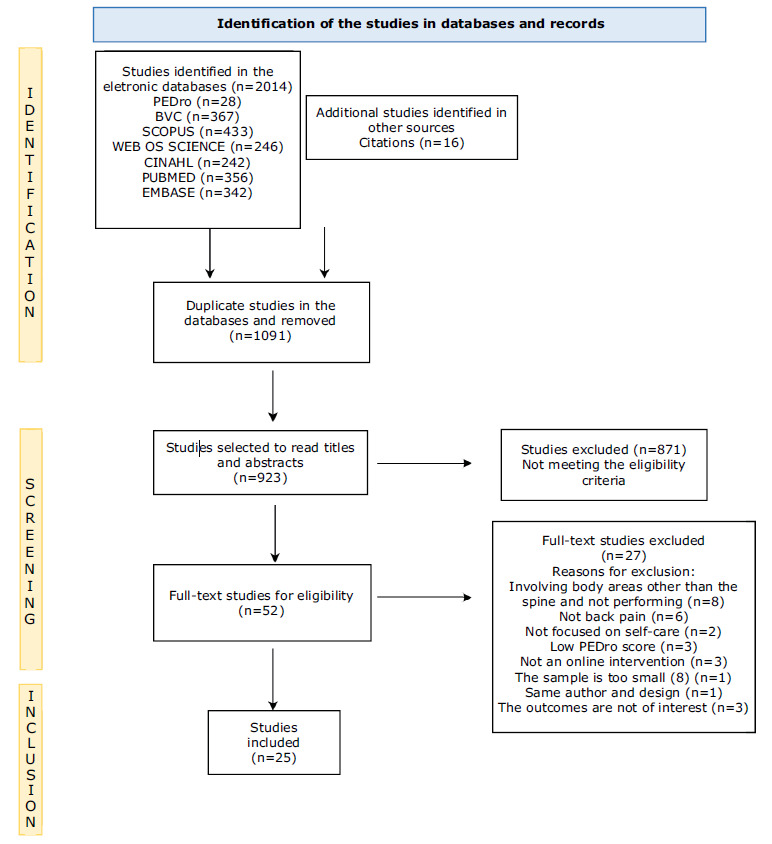
Flowchart corresponding to the studies selected. Florianópolis, SC, Brazil, 2022

A total of 2,014 articles were identified in the databases and, after excluding duplicates, 923 potentially eligible publications were selected for inclusion in this review. A total of 25 studies were selected at the end of the process. The flow corresponding to selection of the articles and the reasons for exclusion are presented in the PRISMA diagram ( Figure 2). The Kappa test showed that there was moderate reliability among the observers (k=0.423; p<0.000; agreement=75%), although there was reconciliation of the divergences. 

**Figure 3 -:** Results of pain intensity and functional disability, sample size, PEDro score and quality classification(25). Florianópolis, SC, Brazil, 2022

**Study**	**Sample**	**Instruments and results**	**PEDro** * **score and** **quality classification**
1 Toelli, et al., 2019 ^( 2)^	94	Numeric Pain Scale (↔) ^†^ Hanover Functional Ability Questionnaire (↔) ^†^	5/10 Medium
2 Shebib, et al., 2019 ^( 6)^	177	Oswestry Disability Index +(↑) ^‡^ Analogue Visual Scale +(↑) ^‡^	6/10 Medium
3 Suman, et al., 2019 ^( 7)^	779	Roland Morris Questionnaire (↔) ^†^	7/10 Medium
4 Hodges, et al., 2021 ^( 8)^	440	Analogue Visual Scale (↔) ^†^ Roland Morris Questionnaire (↔) ^†^	6/10 Medium
5 Moessner; Schiltenwolf; Neubauer, 2012 ^( 14)^	45	Roland Morris Questionnaire +(↑) ^‡^ Numeric Pain Scale (↔) ^†^	5/10 Medium
6 Abadiyan, et al., 2021 ^( 15)^	60	Analogue Visual Scale +(↑) ^‡^ Cervical Disability Index +(↑) ^‡^	7/10 Medium
7 Almhdawi, et al., 2020 ^( 16)^	41	Analogue Visual Scale +(↑) ^‡^ Oswestry Disability Index +(↑) ^‡^	6/10 Medium
8 Lara Palomo, et al., 2022 ^( 17)^	74	Roland Morris Questionnaire +(↑) ^‡^ Oswestry Disability Index +(↑) ^‡^ Analogue Visual Scale +(↑) ^‡^	8/10 Medium
9 Zadro, et al., 2019 ^( 18)^	60	Roland Morris Questionnaire (↔) ^†^ Numeric Pain Scale +(↑) ^‡^	8/10 High
10 Amorim, et al., 2019 ^( 19)^	68	Numeric Pain Scale (↔) ^†^ Roland Morris Questionnaire (↔) ^†^	7/10 Medium
11 Petrozzi, et al., 2019 ^( 20)^	108	Roland Morris Questionnaire +(↔) ^§^ Numeric Pain Scale (↔) ^†^	7/10 Medium
12 Garcia, et al., 2021 ^( 21)^	179	Numeric Pain Scale +(↑) ^‡^	6/10 Medium
13 Sandal, et al., 2021 ^( 22)^	461	Numeric Pain Scale +(↑) ^‡^ Roland Morris Questionnaire +(↑) ^‡^	8/10 High
14 Carpenter, et al., 2012 ^( 23)^	141	Questionnaire (Pain) (↔) ^†^ Roland Morris Questionnaire +(↑) ^‡^	5/10 Medium
15 Chiauzzi, et al.,2010 ^( 24)^	209	Brief Pain Inventory +(↔) ^§^ Oswestry Disability Index (↔) ^†^	6/10 Medium
16 Heapy, et al., 2017 ^( 25)^	125	Numeric Pain Scale +(↔) ^§^ Roland Morris Questionnaire +(↔) ^§^	6/10 Medium
17 Ervine, et al., 2015 ^( 26)^	597	Questionnaire (Pain) +(↑) ^‡^ Dartmouth CO-OP ^||^ (functioning, well-being and quality of life) +(↑) ^‡^	6/10 Medium
18 Krein, et al., 2013 ^( 27)^	229	Numeric Pain Scale +(↔) ^§^ Roland Morris Questionnaire +(↑) ^‡^	7/10 Medium
19 Licciardone; Pandya, 2020 ^( 28)^	102	Numeric Pain Scale (↔) ^†^ Roland Morris Questionnaire +(↔) ^§^	5/10 Medium
20 Lorig, et al., 2002 ^( 29)^	580	Numeric Pain Scale +(↑) ^‡^ Roland Morris Questionnaire +(↑) ^‡^	5/10 Medium
21 Iles, et al., 2011 ^( 30)^	30	Specific functional scale +(↑) ^‡^ Oswestry Disability Index +(↔) ^§^	7/10 Medium
22 Pach, et al., 2022 ^( 31)^	220	Numeric Pain Scale +(↔) ^§^	7/10 Medium
23 del Pozo Cruz, et al., 2012 ^( 32)^	100	Correlation between pain, disability, quality of life and progression to chronicity.	7/10 Medium
24 Chhabra; Sharma; Verma, 2018 ^( 33)^	93	Numeric Pain Scale +(↔) ^§^ Oswestry Disability Index +(↑) ^‡^	8/10 High
25 Gialanella, et al., 2017 ^( 34)^	100	Analogue Visual Scale +(↑) ^‡^ Cervical Disability Index +(↑) ^‡^	5/10 Medium

* PEDro = *Physiotherapy Evidence Database*

† (↔) No difference between the groups

‡ + (↑) Positive and significant effects in relation to the Control Group

§ + (↔) Positive and non-significant effects in relation to the Control Group

|| Dartmouth CO-OP = *Dartmouth Primary Care Cooperative Information Project*

### Evaluation of the quality of the studies

Internal validity and methodological quality by the PEDro scale showed predominance of 21 (84%) medium-quality studies (scores 5 to 7) and 4 (16%) high-quality studies (scores 8 to 10). The mean score was 6.4 (SD: 1.04) out of a total of 10 points ( Figure 3).With the exception of manuscript ^( 14)^ , evaluated independently by the researchers, the scores were directly extracted from the PEDro database. The least met criteria were the following items: concealed allocation of the subjects, blinded subjects, blinded therapist, adequate monitoring and intention-to-treat analysis. Only 10 studies were able to blind the evaluators. 

### Description of the population, interventions and outcomes

The participants were aged 18 years old or more, belonged to the age group from 18 to 65, or were 85 years old with unspecified upper limit. Some considered intermediate ages of 28 to 48 years old ^( 15)^ , 30 to 55 ^( 16)^ , and 30 to 67 ^( 17)^ , and one study included participants aged over 55 years old ^( 18)^ . The mean age in the intervention Group was 49.1 (SD: 7.4). In relation to the participants’ gender, there was 57.9% of women, although 20.8% did not report the percentage of women in the studies. The mean age of the participants was 45.9 years old. Of all 25 studies, eleven reported the participants’ Body Mass Index (BMI) with mean values between 23.2 kg/ m ^2^ and 30.6 kg/m ^2^ in the Intervention Group, with a global mean of 26.89 (SD: 2.09). 

Regarding the design of the studies, all of them were RCTs, published between 2002 and 2022 with predominance of 2019 with 6 (24%) ^( 2, 6- 7, 18; 19- 20)^ and of 2021 with 4 (16%) ^( 8, 21- 22)^ . Of the 25 studies, 9 (36%) were conducted in the United States of America ^( 6, 21, 23; 24; 25; 26; 27; 28- 29)^ , 5 (20%) in Australia ^( 8, 18; 19; 20, 30)^ , 3 (12%) in Germany ^( 2, 14, 31)^ , 2 (8%) in Spain ^( 17)( 32)^ , and 1 (4%) each in several countries such as India ^( 33)^ , Netherlands ^( 7)^ , Iran ^( 15)^ , Jordan ^( 16)^ , Italy ^( 34)^ , Denmark and Norway ^( 22)^ . The overall total was 5,142 participants, varying from 30 to 779 subjects in each study; 9 (36%) studies had sample sizes smaller than 100 participants ^( 2, 14; 15; 16; 17; 18- 19, 30, 33)^ . 

Regarding the digital therapeutic technologies, 9 (36%) used smartphone apps ^( 2, 6, 15- 16, 19, 22, 26, 31, 33)^ , 9 (36%) resorted to websites and online programs ^( 7- 9, 17, 20, 23- 24, 27- 28, 32)^ , 2 (8%) employed telephones ^( 25, 30)^ , 1 (4%) chat discussion ^( 14)^ , 1 (4%) email ^( 29)^ , 1 (4%) virtual reality ^( 21)^ , 1 (4%) Telemedicine ^( 34)^ , and 1 (4%) video game ^( 18)^ . In general, the interventions involved physical exercises ^( 2, 6- 7, 15; 16; 17; 18- 19, 22, 27, 31; 32; 33- 34)^ , education ^( 2, 6; 7- 8, 21; 22- 23, 26- 27, 29, 32)^ and CBT ^( 6, 20- 21, 23; 24; 25- 26, 30)^ . The physical exercises were provided in the form of videos, audios or image-based instructions and could include performance feedback or not. In addition to that, there was technology with sensors to evaluate the activities ^( 33)^ , physical exercises with wearable sensors ^( 6)^ , use of a pedometer ^( 27)^ and tracker of activities ^( 19)^ . The educational material referred to the spine-related pain, and one study was based on the Neuroscience of pain ^( 21)^ . The psychosocial interventions were generally CBT-based and included behavioral strategies, cognitive restructuring, stress management, relaxation, mindfulness and coping practices. In relation to the theoretical grounds of the interventions, 12 (48%) implemented their treatments with evidence-based principles. 

Regarding the spine region affected, 22 (88%) studies investigated pain in the low back area, with 90.9% prevalence of chronic pain; 1 (4.5%) study evaluated subacute non-specific low back pain ^( 32)^ , 1 (4.5%) low back pain of any duration ^( 8)^ and, in 3 (12%) studies, the pain was in the cervical region ^( 15, 31, 34)^ . Although it has been considered as chronic low back pain in this review, the studies ^( 7, 22)^ failed to clarify if all the participants had nonspecific chronic low back pain. The most common instruments for pain were VAS and NRS. One study reported use of the Brief Pain Inventory ^( 24)^ and another one employed a questionnaire with pain frequency, intensity and duration ^( 26)^ . The questionnaire that was most used to assess functional disability was the Roland Morris Disability Questionnaire, followed by the Oswestry Disability Index. 

To facilitate changes in behavior and provide better guidance, the interventions were supported by strategies to increase adherence and monitoring, such as a definition of goals ^( 6, 22, 24, 27, 30, 33)^ , social networking platforms for social support ^( 2, 7)^ , educational messages ^( 16, 22)^ , record of activity levels ^( 2, 18)^ , reminders to perform exercises ^( 16, 22, 31, 33)^ , personalized exercise recommendations ^( 2, 17, 19, 27)^ , motivational messages ^( 8, 27)^ , monitoring of symptoms ^( 6, 14- 15, 19, 24; 25- 26)^ , correct posture reminders ^( 15- 16, 20, 24, 32)^ , exercises with animation and audio ^( 21, 23, 29)^ and reward systems ^( 22, 23)^ . Not all the articles mentioned the engagement level or had interventions to support decision-making. In relation to the adverse events, most of the interventions did not present adverse effects or such effects were not reported by their authors. They were mostly related to increase in pain with physical exercise ^( 25, 27)^ . More musculoskeletal than cardiovascular events were reported, without evidence of excessive harms ^( 27)^ . Some 

participants reported short-term mild or moderate pain associated with physical exercise ^( 20)^ . 

Pain and intensity were measured simultaneously in 19 studies ^( 2, 6, 8, 14; 15; 16- 17, 19- 20, 22; 23; 24; 25; 26; 27; 28- 29, 33- 34)^ , 2 only evaluated pain intensity ^( 21, 31)^ and 3 only assessed functional disability ^( 7, 30, 32)^ . They were not the primary outcomes in some studies. Many other results measures were also evaluated, such as self-efficacy ^( 18, 20; 21; 22; 23- 24, 26- 27, 29- 30)^ , quality of life ^( 2, 7- 8, 15; 16- 17, 22, 25- 26, 32)^ , search for care ^( 18- 19, 29)^ and pain-related beliefs ^( 7, 24)^ . The monitoring period varied from 1 to 12 months. 

In relation to the pain intensity and functional disability outcomes, the comparison between the groups revealed statistically significant improvements (p <0.05) in 54% (12/22) in the pain levels and 47% (10/21) in functional ability of the Intervention Group. There were also additional results that presented significant differences between the groups in favor of the Intervention Group, such as physical activity ^( 19, 33)^ , well-being and quality of life ^( 16- 17, 21, 16)^ , self-efficacy ^( 18, 22, 29)^ , pain-related beliefs ^( 7)^ , decrease in the intention of surgery ^( 6)^ , resistance and posture ^( 15)^ , low back flexion mobility ^( 17)^ , and improved quality of the treatment choices ^( 8)^ . In some trials, the effects disappeared during the monitoring period ^( 8, 23, 27)^ and there was a high follow-up loss rate ^( 7)^ . 

### Qualitative synthesis

Of the 25 articles selected for the review, 19 were included in the meta-analysis and six ^( 14, 18, 21, 25, 29- 30)^ were excluded because it was not possible to group the most specific interventions. The subgroups assembled were related to the type of technology employed and to the spine area affected (cervical or low back). Of these, three articles ^( 16- 17, 19)^ were also excluded in the sensitivity analyses due to the small sample size criterion. Only four articles (16%) ^( 14, 25, 28- 29)^ failed to present the SD. The authors were contacted via email but only one of them answered. There was no influence on the results because the SD was estimated by means of the standard error or the confidence interval, or extracted from graphs. The symmetry of the funnel chart was evaluated visually and proved to be favorable to an improbable publication bias. In the sensitivity analysis, one trial ^( 6)^ was found as a potential heterogeneity source, probably due to sample recruitment in which there was a large difference in the number of participants between the intervention and control groups. In general, there was not significant heterogeneity across the subgroups. When the effect model was changed to the fixed-effect model, the effect size was not significantly different from the results of the random effect modality, which indicates stable results. 

**Figura 4 -:**
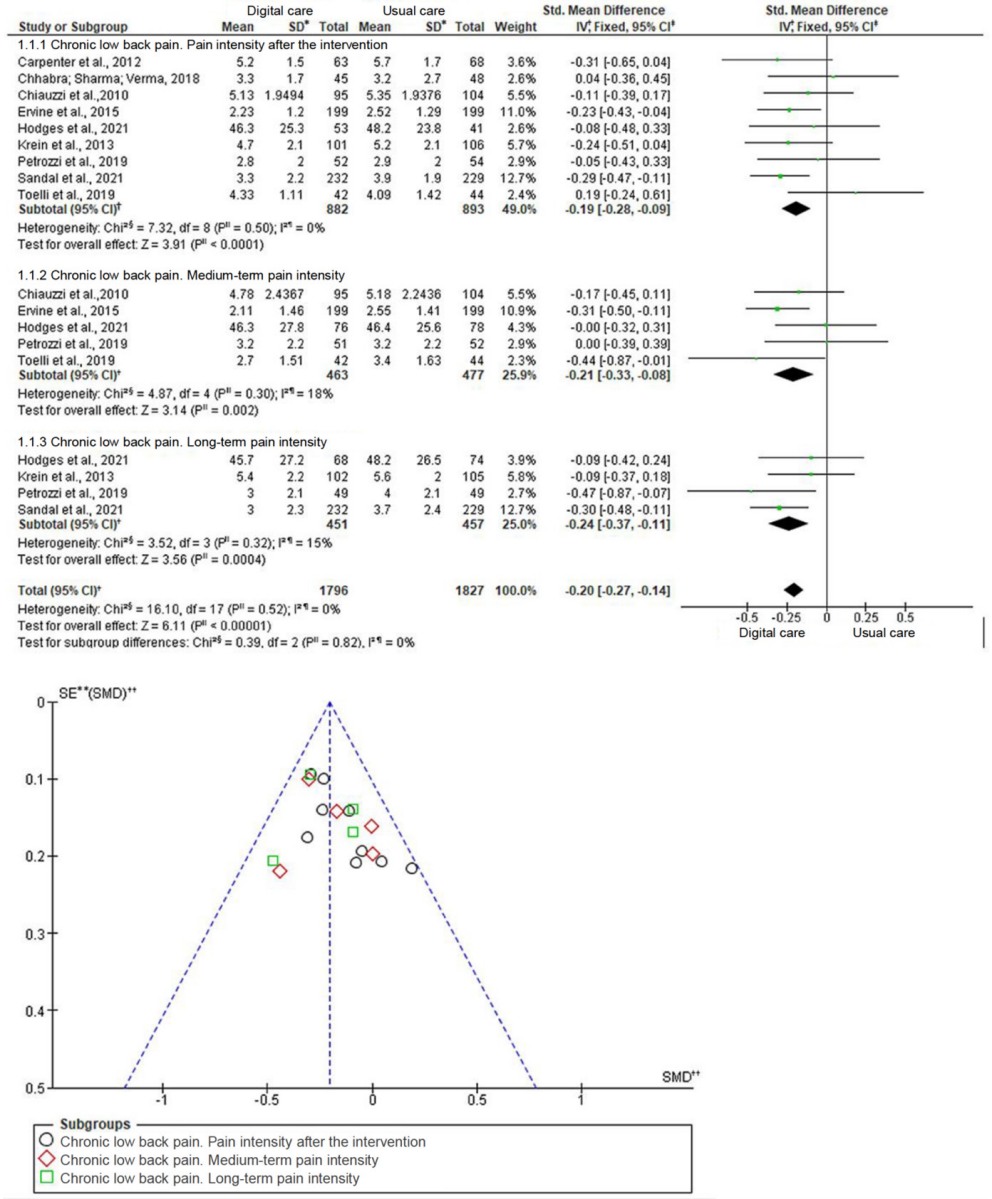
Forest plot and funnel chart corresponding to pain intensity. Florianópolis, SC, Brazil, 2022

Regarding pain intensity in chronic low back pain, the meta-analysis results were classified into three moments: post-intervention, medium-term monitoring of three to six months, and long-term monitoring of nine to twelve months. The first result measure of the RCTs was considered after the intervention. The results showed that digital care was more effective in reducing pain, with a significant and small effect when compared to the Control Group after the intervention [SMD= -0.19, 95% CI (-0.28, -0.09 ), p<0.0001]; statistically significant and moderate effect in the medium term [SMD=-0.21, 95% CI (-0.33, -0.08), p=0.002] and in the long term [SMD=-0.24, 95% CI (-0.37, -0.11), p=0.0004] ( Figure 4). Subgroup analyses were performed to compare the intervention delivered through apps or websites/online programs on pain intensity. Some trials showed a significant and moderate effect [SMD=-0.21, 95% CI (-0.33, -0.10), p=0.0003] with the apps and a significant and small effect [SMD=-0 .16, 95% CI (-0.30, -0.0003), p=0.02] with interventions through websites and online programs in relation to the Control Group with low heterogeneity (I ^2^=0%, p=0.64). Only three studies were related to cervical pain. Due to the high heterogeneity level (Chi ^2^=17.57, I ^2^=89%, p=0.0002), no global effect was calculated. 

**Figura 5 –:**
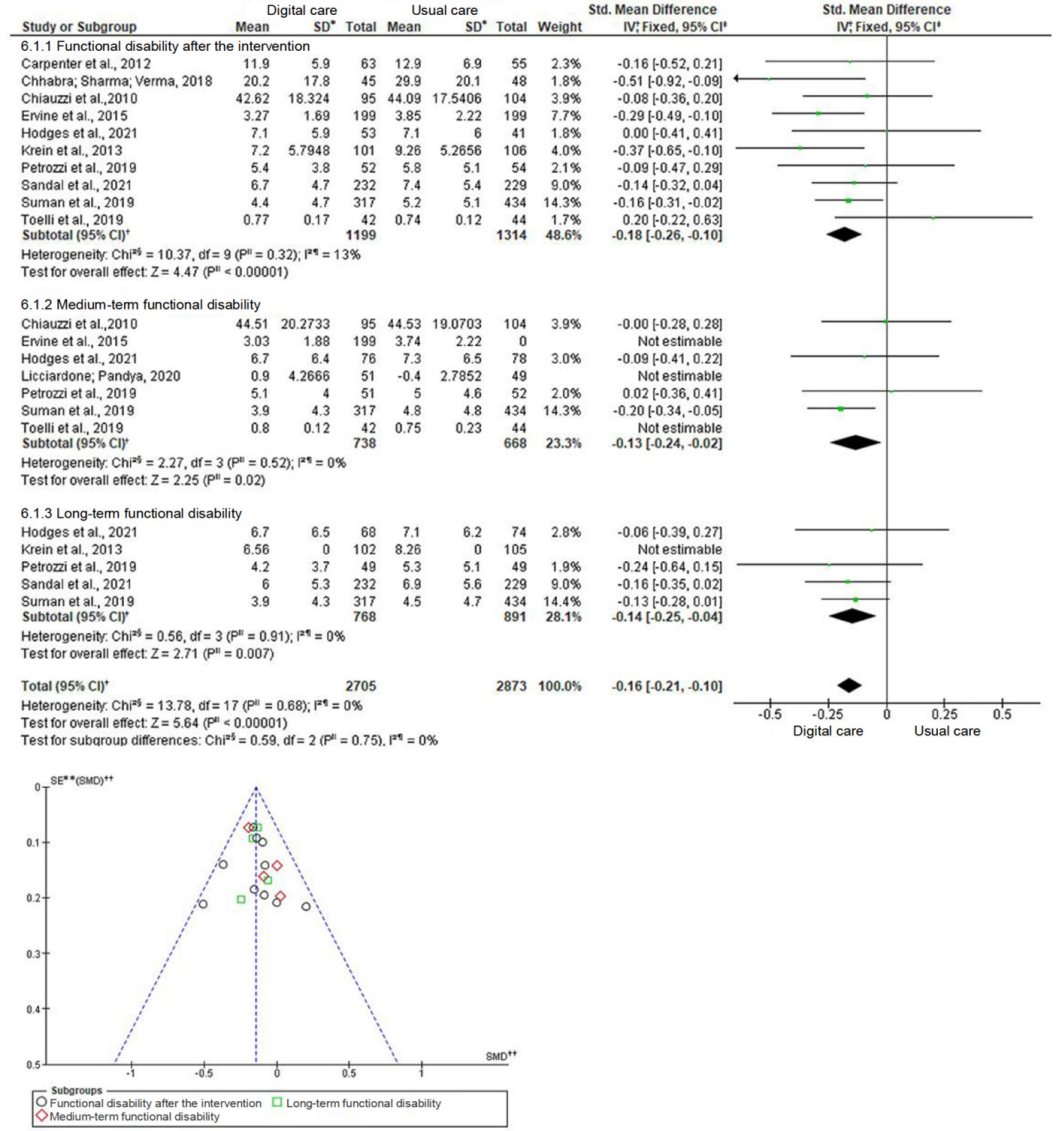
Forest plot and funnel chart corresponding to functional disability. Florianópolis, SC, Brazil, 2022

Regarding disability in low back pain, the results showed that digital care was more effective in reducing functional disability, with a significant and small effect when compared to the Control Group after the intervention [SMD=-0.18, 95% CI (-0.26, -0.10), p<0.0001]; in the medium [SMD=-0.13, 95% CI (-0.24, -0.02), p=0.02] and long [SMD=-0.14, 95% CI (-0.25, -0.04), p=0.007] terms ( Figure 5). When comparing the tests that measured disability using the Roland Morris or Oswestry instruments, a moderate and significant result was found when compared to the Control Group [SMD=-0.24, 95% CI (-0.43, -0.06), p=0.010] in trials that used Oswestry and a small, significant result [SMD=-0.19, 95% CI (-0.29, -0.10), p<0.0001] in those using Roland Morris, with low heterogeneity (I ^2^=18%, p=0.28). Regarding the apps in functional ability, the result was significant with a moderate effect [SMD=-0.21, 95% CI (-0.33, -0.10), p=0.0002] and, regarding the websites and online programs, a small and significant effect [SMD=-0.19, 95% Ci (-0.30, -0.09), p=0.0002] was found, with low heterogeneity (I ^2^=28%, p=0, 18). 

*SD = Standard Deviation; †IV = Inversion of Variance; ‡95% CI = 95% Confidence Interval; §Chi2 = Chi-square test; ||p = Significance level; ¶I2 = Higgins Heterogeneity Coefficient; **SE = Standard Error; ††SMD = Standardized Mean Difference

*SD = Desviación Estándar; †IV = Inversión de varianza; ‡95%CI = Intervalo de Confianza del 95%; §Chi2 = Prueba de Chi-cuadrado; ||p = Nivel de significación; ¶I2 = Coeficiente de Heterogeneidad de Higgins; **SE = Error estándar; ††SMD = Diferencia de Media Estandarizada

## Discussion

This review studied the effectiveness of digital interventions on the management of pain and functional disability in people with spine musculoskeletal disorders. It showed a beneficial result in pain intensity and disability for chronic low back pain with small to moderate effects. It was not possible to determine with certainty the effects over time, either because of the absence of long-term monitoring or because of the decreased effects over time. There was variation in relation to the characteristics, duration, components and support strategies. It was also not possible to determine the impact of the individual intervention components because they offer many combinations and can act independently or synergistically, making it difficult to determine which strategy was responsible for the effect.

According to a cohort study, digital health has the potential to improve the results with increased patient engagement and as a complementary therapy to the clinical practice ^( 35)^ . An SR supports digital care as an additional tool to traditional care, although more evidence of long-term effects is required ^( 36)^ . Another SR found moderate to low evidence that digital programs play a positive role in pain intensity and short-term disability, although there was no evidence for sustained effects ^( 4)^ . Clinical benefits were shown through an app for low back pain, but its methodological quality assessment revealed moderate to high risks of bias, especially in non- randomized trials ^( 37)^ . However, other reviews showed that no intervention was inferior to the Control Group ^(38-39)^ and no study reported adverse effects ^( 38)^ . 

The programs presented variation in their adherence rates. Consequently, it was not possible to establish a relationship between results, adherence levels and factors that led to dropouts due to the several outcomes evaluated and lack of data in the reports. Another review also did not find any evidence in the interventions regarding the decision-making support strategies due to the unclear correlation between user retention and improvement in the primary outcomes, making it difficult to determine aspects of the intervention such as duration or intensity. It would be important to use standardized metrics to ease the comparison ^( 37)^ . 

On the other hand, support interventions favor adherence and the engagement level and can be a key point for the success of these technologies. Therefore, how to attract users has become an important issue for designing online strategies. Some principles such as saving time, interest and information sharing are strongly recommended for designing platforms and increasing engagement ^( 4)^ , particularly if it is a recommendation system based on more advanced data such as machine learning that achieves a sustainable change in behavior ^( 37)^ . 

The meta-analysis showed a greater effect on the functional disability assessment with ODI than with RM. Both tools show reliability and validity with good psychometric properties and ease of use ^( 40, 41, 42)^ . This standardization of measurements facilitates the comparison between the studies and the conduction of a SR. 

The current SR showed interest in the health area towards smartphone apps, m-Heath, for the management of mainly chronic conditions with varied and promising results and a slightly greater effect when compared to “web-health” programs in the meta-analyses. Apps can provide customized health promotion, good user acceptability and easy usability that facilitate self-management ^(16,43)^. The important thing is using these technologies for changes in behavior ^( 19)^ . The potential to improve results and reduce costs is of interest to health system managers and funders ^( 44)^ . However, their efficacy and clinical benefit are still not well proven and require criteria, quality standards, effectiveness and evidence-based content ^( 44)^ . A review showed that the overall quality of the apps is quite low and that they lack valid outcome measures ^( 45)^ . 

Among the contributions of this review we highlight the inclusion of RCTs in which apps were developed by health professionals based on scientific evidence and guidelines and which were evaluated for their effectiveness. Efficacy and usability should always be analyzed before recommending these apps to the patients ^( 16)^ . In this sense, design resources must be considered to enhance efficacy and the standards need to be effectively determined ^( 44)^ . However, some factors are involved in app evaluations. There is a combination of content, platform and interface attributes that hinders determining whether the benefits are the result of specific components or of the app as a whole. Research studies are also limited because apps can be released, updated, modified or removed by developers in the middle of an ongoing survey, rendering the results obsolete; results of new studies may modify the evidence on which the tool is based, making it not valid; and the apps investigated may not be the same as those presented commercially ^( 44)^ . In addition to that, most apps are not scientifically evaluated before they are released to the market ^( 46)^ . 

Exercise was the most prevalent component in the apps. Home-based exercise can prevent recurrent cases, avoid geographic and transportation barriers and financial constraints, and reduce the need for continuous contacts with health professionals ^(15,33)^. An important factor is to adapt the exercise to each user’s subjectivity when considering the patients’ preferences ^(6,19,22,33)^. Favoring a patient-centered approach and their goals and preferences can increase adherence ^( 47)^ . In this sense, technology assists in motivation and in greater involvement with the exercises ^( 48)^ . 

Other RCTs included delivered interventions through online programs, websites, e-interventions and “web-health”. These programs are considered a promising innovation. They reduce the demand for health resources, as individuals manage different components of their own health and enhance their functional independence and self-care ^( 17)^ . They are viable and economical strategies with few or no side effects, and can be accessed at any time and place ^( 23)^ . The COVID-19 pandemic also generated certain interest in home-based self-care ^( 21)^ . However, these programs present barriers related to implementation, such as lack of trained personnel, accessibility and availability ^( 24)^ . There is a need for a website with a comprehensive approach that provides reliable information tailored to the consumer and developed by health professionals ^( 49)^ . 

Another point to consider in these programs is user acceptance and adherence to the treatment to avoid unsustained improvements that occurred in some RCTs. Additional strategies must be implemented to keep people active and engaged and, thus, avoid reductions in the effects over time ^( 27)^ . Sustained improvement may require sustained engagement with the website ^( 8)^ . Evidence was also found that the decision-making support interventions benefit engagement and the self-management process ^( 37)^ . 

Studies with “web-health” programs require other precautions such as adequate recruitment and outcomes since, in some RCTs, the results were small due to the characteristics of the population under study. In this sense, it is advisable to choose criteria that are better established for pain since, for those with no pain at the beginning of the intervention, efficacy in pain intensity or functional results is not expected ^( 7)^ . On-site interventions may be more effective in specific demographic or functional subgroups, such as patients with higher pain levels ^( 24)^ . In the RCT, the improvement was higher for those individuals who reported moderate to severe levels of pain-related disability at the beginning of the study ^( 27)^ . Screening is also advisable, which consists in classifying the patients according to their signs and symptoms to prescribe certain exercises and other effective self-care options ^( 17)^ . The content of the program must be specific and sufficient for the profile of the population ^( 20)^ . 

Combining domains such as function, disability and health would be a useful and valid structure for setting goals in musculoskeletal conditions ^( 45)^ . As pain is biopsychosocial in nature, a multimodal approach should be person-centered, adapted to the individual’s preferences and attitudes, enable long-term personal control over the symptoms, lessen the need for supervision, and be evidence-based ^(45,50)^. These self-care allies are key components of a digital program ^( 50)^ . The programs need to capture the full multidimensional and biopsychosocial nature in the therapeutic processes ^( 51)^ . 

Other RCTs evaluated other technologies such as Virtual Reality (VR), video games, other ways of delivering the intervention such as telephone, chats and email messages, which may be appealing to the users. The VR treatment offers immersive 3D experiences with stereo sounds and elements such as rich colors and scenic environments that adapt to specific conditions such as pain in the therapeutic context ^( 21)^ . Video games applied to aged participants with chronic low back pain showed good results ^( 18)^ . It is noted that some results had small effects, but the strategy options show promising results. Whether due to high adherence, interactivity or motivation ^( 18, 21, 25)^ , the possibility of association with other therapies ^( 18)^ , remote monitoring ^( 34)^ , improved self-efficacy ^( 18. 29)^ , easy-to-execute programs ^( 34)^ with adequate control group ^( 21)^ can favor self-management, reduce the health care use and ease adherence to home-based exercises. The cost-benefit ratio depends on each patient’s circumstances and preferences. The practicality of the non-face-to-face intervention can be feasible, effective and well tolerated ^( 25)^ . 

Finally, digital interventions have the potential to offer safe, high-reach, low-cost, readily accessible and scalable practices, and they favor access to health care in a non-face-to-face way to more people ^( 38)^ . In addition to that, monitoring their condition and goals exert an influence on their cognitive, emotional and behavioral response to pain ^( 4)^ . 

As for the limitations, in the first place, the interventions used several technologies, outcomes and outcome measures. The subgroup analysis ensured better homogeneity in the meta-analyses. There is a need to standardize the reporting of results in clinical trials of patients with non-specific low back and cervical pain. Most of the studies were related to low back pain and few dealt with the cervical region. Trials with small samples of less than 100 participants were included, which can reduce statistical power, as well as others with non-comparable control groups that had face-to-face self-care interventions, different observation periods and lack of long-term monitoring, making it difficult to assess sustainability of the results. Some lacked more clearly defined inclusion criteria, leaving doubts about whether or not the participants’ pain was chronic. In addition to that, pain intensity and functional disability were not the primary outcomes in all the studies. Also, data such as duration and intensity of intervention or best decision-making support strategies could not be extracted. In addition, most of the studies were of moderate quality. Therefore, the results should be analyzed with caution.

## Conclusion

In this review, digital care interventions showed a beneficial result in reducing pain intensity and functional disability mainly for chronic low back pain, with small to moderate effects. The comparison between the groups revealed statistically significant improvements in half of the studies in the pain levels and in slightly less than half in terms of functional disability in the Intervention Group, when compared to the Control Group. In this sense, it can be asserted that digital care is promising to support the self-management of spine musculoskeletal conditions. Additional research studies with more standardized outcomes, sample sizes and adequate control groups are required to ease the comparison and search for evidence.
